# Students’ perception and learning on case based teaching in anatomy and physiology: An e-learning approach

**DOI:** 10.30476/jamp.2020.87332.1304

**Published:** 2021-01

**Authors:** NEERAJ VEDI, PUJA DULLOO

**Affiliations:** 1 Department of Anatomy, PSMC, Karamsad, India; 2 Department of Physiology, PSMC, Karamsad, India

**Keywords:** Teaching, Leadership, Self-directed learning, Medical student

## Abstract

**Introduction:**

Case based teaching (CBT) has been accepted as an effective interactive learning strategy. Digital portals allow the students to learn the content at their own pace, explore various resources and finally enable them to discuss within group and build team work approach, which is a prime focus in the health care professional field. The aim of this study was to assess the perception and learning outcome of first year medical students towards CBT using e-learning approach.

**Method:**

This is a non-randomized, interventional study on first year undergraduate medical students from 2017-18 batch (43) and 2018-19 batch (41) of Sumandeep Vidyapeeth University. They were divided into a group of 8-10 members, who attended six sessions of case-based teaching via Google group. Learning outcome was analyzed by comparing the students who participated in the sessions and those who did not. Feedback survey questionnaire was analyzed by Mann Whitney ranking test and focus group discussion by thematic analysis for qualitative analysis manually.

**Result:**

A p value <0.01 was considered statistically significant for post-test by e-learning tool for CBT. Participants agreed that CBT is a good way to conceptualize applied aspect of basic science, enhance critical thinking, and explore varied resources. Thus, they confirmed that team building approach and leadership qualities for managing the group contributed to better understanding of the course and would be useful to them in near future.

**Conclusion:**

Usage of Google group technology for CBT allowed medical students to explore clinical application of basic sciences course from the first year of the program, going beyond the classroom, thus developing self-directed learning and team building approach.

## Introduction

The Indian medical curriculum is governed by the structural curriculum provided by the Medical Council of India (MCI). After two decades, the curriculum has been restructured from traditional discipline based on competency driven curriculum ( 1 ). However, prior to this restructuring many pre-clinical faculties of the medical institutes have been introducing various interactive techniques like that of case-based learning (CBL). The researchers in the basic science course have concluded that case-based teaching (CBT) stimulates the students to apply cognitive skill as per clinical context ( 2 ), and develops and improves complex-problem solving skills ( 3 , 4 ), thus emphasizing the patho-physiological basis, in clinical context. Researchers have identified that usage of actual real-world clinical cases in CBT allows the students to reach a correct diagnosis and recognize the importance of inter-relatedness, ( 2 ) by linking the learned concepts ( 5 ). Moreover, researchers have identified that CBL provides better opportunity to students for the usage of different resource materials and have better interaction with their peer and instructor ( 6 ). Authors have reached various conclusions as per score acquired after the end of the CBT course; some found enhanced performance using lectures ( 7 - 9 ), whereas others concluded to have better performance with CBL ( 10 - 13 ). Moreover, some showed no differences between the two methods ( 14 - 16 ). Studies differ in results as compared to the implementation of the CBL pedagogy, group size, and many other factors which might lead to variations in the outcome of the process ( 3 , 17 ).

Developing countries, like India, have limited human and infra-structural resources facilities for teaching medical students; thus, there is a need and requirement to look for free portal which would be easy to work on not only for the faculty members, but also for undergraduate students. 

Web-based learning activities are considered independent, active learning processes, allowing large group of learners together as ‘virtual’ group ( 18 ); however, they cannot replace the traditional teaching strategies in medical educational setting ( 19 ). The digital approach of learning may not be superior to traditional method, but it has a great potential to motivate the students towards self-directed learning ( 18 , 20 ). Presently, all the Indian medical institutes have informational websites, as per requirement by the MCI, although the usage of the technology for teaching-learning aspect is still awaited in the majority of medical institutes. Google groups have been used for various medical faculty development programs within the country, India, like Advanced Course in Medical Education (ACME); thus, they can be used as one of the methods for students’ learning.

At our institute, we introduced a voluntary optional teaching learning approach using Google group, as an e-learning tool, for first year undergraduate medical students from August 2017 to July 2018 (2017-18) batch and August 2018 to July 2019 (2018-19) batch. We aimed to expose those pre-clinical students to prospective real life situation for physiology and anatomy subjects by contemporary case-based learning method. The perception and learning outcome of those students were assessed for this newer approach of teaching learning strategy.

## Methods

This non-randomized interventional study was conducted on undergraduate medical students from two consecutive batches 2017-18 (Academic year August 2017 to July 2018) and 2018-19 (Academic year August 2018 to July 2019). The purpose of the study was explained to each batch of the students. 43 students from 2017-18 batch and 41 from 2018-19 batch, out of 150 students from each year batch, volunteered to participate in the study. The students who volunteered to participate were considered as the experimental group (Group-B) and those who did not participate were enrolled as the control group (Group-A).

The experimental (Group-B) undergraduate medical students from each year batch (2017-18 & 2018-19) were again explained about the aim, purpose and methodology of the study and written consent was taken. The voluntary participants were divided into groups (8-10 students per group). All the groups were requested to prepare an independent Google group, including the principal investigator, concerned faculty member, and student group members, so that they were able to work online.

### On-line CBL for participatory/experimental group

One of the students within the group was chosen a leader, either by the group members or by the instructor, blindfolded. The group leader was in touch with the instructor via e-mail only. There would be no in-person communication between the instructor and the groups. In case any student within the group had issues, he/she could contact the instructor independently via email or in person, if required. The instructor had the flexibility to provide guidance to the leader in the case of any serious issue.

Based on the topics covered via conventional lecture series, for the concerned batch, for anatomy and physiology course, a clinical paper case was prepared by the instructors and relevant application-based questions were framed integrating the basic science courses horizontally and vertically. This was sent to each group by the instructor. All the groups received the same case for the discussion. A specific timeframe was laid by the instructor for the completion and submission of the task. 

Attending the conventional lecture for the topic was not mandatory for the participants to join the e-learning Google group. 

### Role of the leader

The group leader was there to share the cases within the group, as a soft and hard copy and motivate the group members to participate in the discussion by finalizing the venue and time for face to face or online discussion, so as to reach the final outcome of the case. The leader also initiated the discussion in the framed group and compiled the final response to each question as agreed and approved by the group members, after using various resources at their own level (self-directed learning approach). At the end of the activity, the leader uploaded the answers for the questions on the framed Google group. A feedback response was provided by the instructor for each clinical case after submission by all the group leaders. Group leader also intimated the instructor about those members who failed to participate.

With every new clinical case, a new group was formed, with a new student as the group leader. This imparted leadership qualities and developed the team building skill for the discussion within the group members. A total of 4 sessions with such methods were conducted for 2017-18 batch, while only 2 sessions were conducted for 2018-19 batch.

Participants had the openness to withdraw from the study without seeking permission from the instructor. In case they wanted to rejoin the group for subsequent cases, then as a prerequisite instructor’s permission was required.

### Learning analysis

After every case, a test was given to all the students of the class for the topic and learning outcomes of the two groups, participant- Group-B (43 from 2017-18 batch & 41 from 2018-19 batch) and non-participant- Group-A (107 and 109 from respective batches), were compared.

On completion of the test, the cases were openly discussed within the class for better understanding of every student enrolled in the course. This motivated some more students to participant for the research study. After the first case discussion, the participants increased from 15 to 43 in 2017-18 batch, while in 2018-19 batch it increased from 25 to 41.

### Quantitative and qualitative assessment

Data related to perception of students for corrective implementation and importance of online CBT were collected using a survey questionnaire, having open-ended and closed ended questions, based on three-point Likert’s scale, after getting it validated by subject experts. The readability index had the text for the average grade level of about 6 (easily understood by a 11 to 12 year old one). The content validity ratio (CVR), after inputs from the six qualified experts for each item for accuracy, items as per objective of the study and grammatical correction was +1 for 19 and 0.61 for 6 items, thus having the content validity index (CVI) 0.88 ( 21 ). A pilot test was done for those survey questions on 2016-17 batch (N=10) first year undergraduate students, who had undergone a similar process on voluntary basis. The Cronbach’s Alpha (CA) value for those 25 items for these students was 0.86.

A focus group discussion (FGD) guideline with questionnaire was developed and validated (CVI-0.941) for identifying the perceptive of participants regarding the tool. Prior to initiation of FGD, participants were explained that the research investigators were interested in their truthful feelings and attitudes towards the teaching approach. Two such FGDs were facilitated, each having 8 to 10 students with 4 elements to make sure that students were comfortable and relaxed. During the first welcoming element, the participants were familiarized with the FGD, highlighting the ground rules followed by warm-up element by introducing to the moderator, and each member, so each individual participant had time to express his/her thoughts and was encouraged to share different points of view by the use of two essential techniques, “the pause and the probe” ( 22 ). Questions were specific, yet open-ended, and additional questions were allowed to emerge within the context of the conversation. For example, one of the general questions was, “How online case-based learning session worked for you?” Probing questions then allowed the participants to expand on their responses, like “Can you please elaborate it with an example?”

Informal member checking and summarizing the content was used throughout the two focus groups to ensure that students’ responses were correctly interpreted ( 23 ). Focus groups ranged in length from 40 to 45 minutes. Focus groups were recorded using an audio recording device and transcribed by the author, word by word, which was validated by an external and internal expert manually.

Pre- and post-test were done based on multiple choice questions (MCQ) to assess the learning outcome of the students. Statistical analysis of the data for frequency distribution, paired t-test and Mann Whitney ranking test, was done to identify the mean and standard deviation of the response and ranking for each question as per test in both batches of students. Data for FGD was deductively analyzed manually using Braun and Clarke's (2006) 6-step thematic analysis, ( 24 ) starting from becoming familiar with the data, generating initial codes, followed by searching for themes, reviewing the themes, defining the themes, and lastly writing them. 

### Ethical Approval

The study was commenced after approval from Sumandeep Vidyapeeth Institutional Ethics Committee (SVIEC/ON/Medi/RP/18002; 5^th^ February 2018).

## Results

The quantitative data are analysis and represented in the form of tables, while the qualitative analysis is represented as paragraph under two main themes. Table 1 shows the comparative analysis by pre-and post-test (2017-18 batch) for participant/experimental (Group-B) and non-participant/control (Group-A) group of students. The statistical significance (p<0.001) was observed from the pre- and post-test for Group-A as well as Group-B students. The comparative post-test score for the learning outcome between Group-A and B showed statistical significance (p< 0.416). The Cronbach's Alpha Reliability coefficient for 25 perceptional survey questionnaires items (Annexure-1) for the first year undergraduate medical student batch 2017-18 was 0.928 and for those of 2018-19 it was 0.912. 

**Table 1:** Comparative statistics by student’s t-test for the pre- and post-test in non-participatory/control group and the participatory/experimental group of students (2017-18 batch)

Comparative groups		Mean±SD	Difference & t-statistics
Pair 1	Pre test	27.56±10.98	9.640 & 3.955 (p<0.0001)
Non-participant group (Group-A) (N=83)	Post test	37.20±11.87	
Pair 2	Pre test	31.63±10.29	10.15 & 5.836 (p<0.0001)
Participant Group (Group-B) (N=40)	Post test	41.78±12.05	
Pair 3	Post test %-Group-A (N=83)	37.20±11.87	4.58 & 2.058 (p<0.0416)
Post-test between A group & B group	Post test %-Group-B (N=40)	41.78±12.05	

Table 1 shows the comparative analysis of pre- and post-test for participant (B) and non-participant (A) groups of students. The two groups showed statistical significance (p<0.001) by analyzing pre- and post-test as in pair 1 and 2, while the post-test analysis of participant (B) and non-participant (A) groups showed statistically significant results (p<0.0416) for the learning outcome score, as in pair 3. Participants who appeared for both pre- and post-test were only included.

Table 2 shows frequency distribution statistics and Mann Whitney ranking test for the survey questionnaires from two batches. Some rank variability was identified as per the batches although both ranked the highest for question 2.

**Table 2:** Frequency distribution statistics and Mann Whitney ranking test for the survey questionnaires from the first year medical students (2017-18 (N=43) and 2018-19 (N=41))

Survey Question No.	Batch 2017-18		Batch 2018-19	
	Mean±SD	Ranking	Mean±SD	Ranking
1	3.95±0.71	05	4.29±0.78	06
2	4.27±0.74	01	4.59±0.55	01
3	3.32±0.93	25	3.56±0.9	16
4	3.41±0.9	24	3.1±0.97	22
5	3.44±1.1	23	3.12±1.27	21
6	3.73±0.87	13	3.83±1.2	10
7	3.66±1.44	15	3.51±1.36	17
8	3.54±1.0	18	3.51±1.19	18
9	3.66±0.94	16	2.83±1.22	25
10	3.71±1.12	14	3.83±1.30	09
11	4.1±0.74	02	4.32±0.79	04
12	3.8±1.08	08	3.98±1.28	07
13	3.44±1.12	22	3.41±1.22	19
14	4.07±0.79	03	4.51±0.55	02
15	3.93±0.76	06	4.32±0.79	05
16	4.0±0.89	04	4.41±0.59	03
17	3.54±1.0	19	3.66±1.06	12
18	3.88±0.75	07	3.59±1.14	13
19	3.78±1.01	10	3.07±1.19	23
20	3.8±0.72	09	3.56±1.05	14
21	3.49±0.87	21	3.56±0.95	15
22	3.76±0.89	11	3.71±0.96	11
23	3.63±0.92	17	3.07±1.06	24
24	3.73±1.11	12	3.9±1.08	08
25	3.49±1.27	20	3.27±1.48	20

The Cronbach's Alpha Reliability coefficient for 25 perceptional questionnaire items for the first year medical students batch 2017-18 was 0.928, and for those of 2018-19 it was 0.912. Table 2 shows frequency distribution statistics and Mann Whitney ranking test for the survey questionnaires from two batches. Some rank variability was identified as per batche although both ranked highest for question 2.

In order to identify perceptional difference for the study methodology between the undergraduate medical students from two different batches, an independent sample-test was done, as shown in Table 3. Statistical significance for Levene's Test for Equality of Variances was observed for question number 1,18, and 20 between the two batches of undergraduate medical students. A comparative statistical significance (p<0.01) value was observed for question number 6 only in students of batch 2018-19, while no significant difference was observed in 2017-18 batch as per gender.

**Table 3:** Independent unpaired t- test for the survey questionnaires from the first year medical student’s batch 2017-18 and 2018-19

Comparison of the mean for Survey feedback question for Batch 2017-18 & 2018-19		t-test for Equality of Means			Levene's Test for Equality of Variances	
		T	Sig.	Mean Difference	F	Sig.
Q1	Equal variances assumed	-2.112	0.038	-0.339	8.789	0.004*
	Equal variances not assumed	-2.105	0.038	-0.339		
Q2	Equal variances assumed	-2.340	0.022	-0.330	0.026	0.872
	Equal variances not assumed	-2.356	0.021	-0.330		
Q3	Equal variances assumed	-1.068	0.289	-0.212	0.156	0.694
	Equal variances not assumed	-1.069	0.288	-0.212		
Q4	Equal variances assumed	1.705	0.092	0.344	0.034	0.855
	Equal variances not assumed	1.701	0.093	0.344		
Q5	Equal variances assumed	0.954	0.343	0.250	1.478	0.228
	Equal variances not assumed	0.951	0.344	0.250		
Q6	Equal variances assumed	-0.579	0.564	-0.132	1.392	0.242
	Equal variances not assumed	-0.575	0.567	-0.132		
Q7	Equal variances assumed	0.459	0.648	0.139	0.002	0.965
	Equal variances not assumed	0.459	0.647	0.139		
Q8	Equal variances assumed	-0.002	0.998	-0.001	1.406	0.239
	Equal variances not assumed	-0.002	0.998	-0.001		
Q9	Equal variances assumed	3.593	0.001	0.845	3.344	0.071
	Equal variances not assumed	3.569	0.001	0.845		
Q10	Equal variances assumed	-0.745	0.459	-0.201	0.703	0.404
	Equal variances not assumed	-0.743	0.460	-0.201		
Q11	Equal variances assumed	-1.486	0.141	-0.247	1.136	0.290
	Equal variances not assumed	-1.484	0.142	-0.247		
Q12	Equal variances assumed	-0.725	0.471	-0.185	0.647	0.423
	Equal variances not assumed	-0.721	0.473	-0.185		
Q13	Equal variances assumed	0.107	0.915	0.027	0.375	0.542
	Equal variances not assumed	0.107	0.915	0.027		
Q14	Equal variances assumed	-2.826	0.006	-0.419	0.033	0.857
	Equal variances not assumed	-2.848	0.006	-0.419		
Q15	Equal variances assumed	-2.324	0.023	-0.387	2.989	0.088
	Equal variances not assumed	-2.321	0.023	-0.387		
Q16	Equal variances assumed	-2.538	0.013	-0.415	0.073	0.788
	Equal variances not assumed	-2.560	0.012	-0.415		
Q17	Equal variances assumed	-0.553	0.581	-0.124	0.290	0.592
	Equal variances not assumed	-0.552	0.582	-0.124		
Q18	Equal variances assumed	1.435	0.155	0.298	11.303	0.001*
	Equal variances not assumed	1.421	0.160	0.298		
Q19	Equal variances assumed	2.903	0.005	0.694	0.041	0.841
	Equal variances not assumed	2.890	0.005	0.694		
Q20	Equal variances assumed	1.306	0.195	0.253	8.339	0.005*
	Equal variances not assumed	1.294	0.200	0.253		
Q21	Equal variances assumed	-0.368	0.714	-0.073	0.439	0.509
	Equal variances not assumed	-0.367	0.714	-0.073		
Q22	Equal variances assumed	0.068	0.946	0.014	0.091	0.763
	Equal variances not assumed	0.068	0.946	0.014		
Q23	Equal variances assumed	2.480	0.015	0.531	0.011	0.917
	Equal variances not assumed	2.470	0.016	0.531		
Q24	Equal variances assumed	-0.776	0.440	-0.182	0.485	0.488
	Equal variances not assumed	-0.776	0.440	-0.182		
Q25	Equal variances assumed	0.817	0.416	0.243	2.191	0.143
	Equal variances not assumed	0.813	0.419	0.243		

*P value=extremely statistically significant

Table 3 shows independent sample-test for the survey questionnaires from the 2017-18 and 2018-19 batch. Statistical significance for Levene's Test for Equality of Variances was observed for questions number 1,18, and 20 (p<0.05) between the two batches of undergraduate medical students.

A comparative statistical significance (p<0.01) value was observed for questions number 6 only in the 2018-19 batch, while no significant difference was observed in the 2017-18 batch as per gender.

### Data analysis for focus group discussion

The majority of participants in this study agreed with the importance of case based teaching and valued the use of digital technology for better understanding of the cases and topics related to the case. Two focus group discussions were conducted; Group-1 (N=9) had 5 Male (M) and 4 Female (F) participants, out of whom 2 participated with a role of leader (L); while Group-2 (N=8) had 3 Male (M) and 5 Female (F) participants, 4 of them participated as leaders (L) undergraduate first year medical student batch of 2017-18. Other members who were not leaders were in the group as members (NL).

Table 4 shows six themes emerged from the transcript of the two focus group discussion sessions.

**Table 4:** Themes emerged from the two focus group discussion sessions

Theme	Sub-theme	Description
Benefits	Clinical knowledge	*“…Thinking in depth, clinical thinking approach improved....”* (G-1ML)
		*“Early stimulation is good specifically clinically, at least at out 1st year level”* (G-2FNL)
	Leadership quality	*“Leadership skill development, managing everyone at one point of time and making them work. *“ (G-1FL)
		*“..I can understand how participants respond to the task.”* (G-2ML)
	Self-directed learning	*“We as a group explored other resources to understand the case and topic.”* (G-1FNL)
Scope for upgrading	Before the session	*“Brief up of the component should be given.”* (G-1FNL)
	During the session	*“Give 5 questions of previous case before working on new case.”* (G-2MNL)
	After the session	*“Give a reward to the task as grades i.e., some portion of grade percentage should be kept for participating in such an informative sessions.”* (G-2ML)
		*“In person feedback or in class would be good.”* (G-1FL)
		*“Follow up should be by teacher after every case feedback.”* (G-2FL)

## Discussion

The present study shows a statistically significant learning outcome from the CBT compared to that of traditional lecture approach. Our study results are in the same line with Bansal and Goyal ( 25 ), Bennal et al, ( 26 ) and Dulloo and Pathare’s ( 6 ) studies, showing significant learning outcome from the CBT approach and increased academic scores on the topics taught by CBT in the physiology course. The study by Majeed ( 9 ) concluded that test performance of participants was better after didactic lectures (mean, 17.53) rather than after case-based teaching. Researchers have highlighted the popularity for the use of digital platforms for online case based learning ( 27 , 28 ), ensuring students satisfaction ( 29 ); however, some researchers concluded reduction in satisfaction level either due to defect in the learning platform ( 30 ) or poor navigation portals ( 31 ).

The present study showed varied ranking and correlation for each question as per the two batches of first year medical students. The perceptional questionnaire showed that the participants appreciated the time allocated to complete the task, and the opportunity they had for the usage of different resources to accomplish the clinical case task. Bennal et al.’s ( 26 ) study highlights that CBT promotes active involvement, motivates and increases attention span during the lecture session. Moreover, the present study also showed that both batches agreed that CBT posed challenging questions to help them develop analytical and critical thinking which would be helpful for them in near future. Majeed’s ( 9 ) study showed that 65 to 72% of students found that CBT improved their knowledge about the topic better than lectures. Dulloo and Pathare’s ( 6 ) study showed that 75.9% of students accepted CBT method as an encouraging, informative and motivational approach for learning the concepts. Participants of the present study even felt that this type of teaching learning approach was a good way for peer discussion strategy. Dickinson et al.’s ( 32 ) study concluded that CBL increased the students’ engagement in class, depth of discussion within their teams, and depth of discussion between the teams, helping the students to apply basic science concepts to the clinical material and have better understanding of the disease processes as per case, and have authentic learning experience. Moreover, some researchers indicated that CBT method was related to the course content with a clinical situation, thus having an active learning approach ( 32 - 36 ). 

Nicklen et al.’s study concluded that the students accepted web-based conferencing as a suitable mediocre to participate in case-based learning (CBL). Even the participants were satisfied with the learning activity and accepted the flexibility in the program; thus, they were able to meet their learning objectives ( 37 ). The study on computer assisted teaching, by Dulloo ( 38 ), identified the flexibility and repeatability of the topic for better learning and discussion, and even motivated participants for self-directed learning. The participants in present study also felt that their self-directed learning was improved by the online CBT learning process. They mentioned “There is so much knowledge other than books which must be shared. I have learned to explore.” “Searching things in google helped me to increase the knowledge” to show why and how their self-directed learning was increased by participating in this teaching method. Researchers ( 25 , 38 - 42 ) concluded from their studies that the CBT learning increased the team spirit within the small group, developed a sense of healthy competition in between the groups, and increased the collaborative and communication skills within students. However, Abraham et al. ( 43 ) found that students expressed frustration with CBL, including confusion of faculty student expectations, insufficient faculties, student tutorials, self-directed learning strategies, lack of integration into the curriculum, and insufficient time ( 44 ). Wittich et al. ( 45 ) specified quality improvement, by CBT, to prevent patient adverse events.

Researchers also have commented for the faculty perception; they regarded CBT as time intensive ( 46 ), specifically in terms of preparation ( 47 ). Blewett and Kisamore ( 48 ) highlighted the less faculty facilitator requirement in case of online CBL which could be applied to a large group of students and having fewer intra-group problems. Smith and Christie ( 49 ) considered interactive case-based assignments as effective learning tool for inter-professional learning. 

The present study also indicated that this type of online approach had a great potential to develop leadership qualities, specifically from 2017-18 batch by mentioning “managing and coordinating within group members, time-management especially being a group leader” and “interaction with others and knowing new things helped me to work as a team”. Participants from the batch 2018-19 did not show a promising outcome for the development of these qualities although they also mentioned about improving the leadership qualities “confidence of asking confusing questions while dealing with peer” and “cooperation with the team members, self-confidence”. Statistical significance for the responses observed between batch 2017-18 and 2018-19 was only for questions number 1,18, and 20, specifying that all the participants from both the batches agreed that they required this type of online clinical session and it allowed them to have better peer discussion and ability to enjoy working in a group. Our findings are similar to that of Doran et al.’s study which initially identified that students struggled to manage the process of working in groups, but once they started having collaborative work, they regarded the method as a positive feature of their learning experience ( 44 ). However, few researchers found that the learning style of the students did not influence their perceived learning experience with case-based e-learning ( 50 , 51 ).

The present study showed no gender variation as per the perceptional questionnaire in both batches, except for question 6 in 2018-19 batches. Gender variability was not focused by other researchers in terms of perception or learning for CBT. 

The focus group discussions with the participants identified various benefits for the online case- based teaching strategy and identified certain components to work upon; some participants considered it as an extra burden, while few mentioned that students copy-pasted the answer from other students without understanding the basic concept. Some of the participants were not satisfied with the online feedback and suggested to have an in-person or face to face discussion for the cases with the instructor. Some wanted marking system to be introduced for the assigned task. Some asked to have questions related to previous case before starting the next session, while few wanted to have a brief outline of the topic before the case presentation. Thus, there is a scope of improvement by modifying this way of teaching clinical aspect to health professional students during pre-clinical program. 

Ali et al. ( 52 ) developed an interactive CBL System (iCBLS) which was a CBL system which created real world clinical cases with a semi-automatic approach, formulated the summaries of CBL cases and provided feedback for formulated cases. Thus, allowing the students to practice real-world clinical cases before and outside the class can promote learning capabilities; save class time for effective discussion and enhance the academic experience of medical students. Waliany et al. ( 53 ) in their study have concluded that the CBT continues to grow as an instrumental pedagogical model in preclinical education with an objective of imparting real-world clinical cognitive skills. 

The present study used a simple digital technology for CBL for students of pre-clinical program which requires minimum human resource and low cost technology to enhance not only clinical approach of students, but also makes them lifelong learners by directing them towards self-directed learning. 

### Limitations

This study had several limitations. First, the study was done in one institute, although for two batches, but with few clinical cases and few voluntary participants. The impact of this short-term strategy may be limited. The learning outcome for the batch 2018-19 was not identified and the participant group size was less than that of non-participant group. The impact on long-term change in behavior and learning outcome would take a longer time span to be identified. Extensive data would be better for statistical results. Faculty perception was not taken into account, which might have added value to the research study. There is a strong need to motivate non-participant students, so that non-bias environment will be acquired.

## Conclusion

The old would forever be gold, whilst the new shines brightly like diamond but deprives the forge or shade. They are just rocks and stones. Change is necessary to rejuvenate the traditional Gurukul method by having practical approach to the topics taught while associating digital portals for various learning strategies, like case-based approach. It is concluded that online CBT adds to the students’ interest and inclination for enhancement of critical analysis approach and covers all the dimensions of medicine field, at their pace, without disturbing the routine formal classes. Moreover, google group is an easy, cost effective tool, which can be used by a large number of students with a single facilitator. Thus, the results can be well used by institutes having limited human and infrastructural resources, like that of India and other under-resourceful countries. 
